# Covid-19 pandemic and risk of Myopia

**DOI:** 10.1016/j.amsu.2022.103944

**Published:** 2022-06-03

**Authors:** Rozhan Khezri, Fatemeh Rezaei, Fateme Darvish Motevalli

**Affiliations:** Department of Epidemiology, School of Public Health, Iran University of Medical Sciences, Tehran, Iran; Department of Social Medicine, Jahrom University of Medical Sciences, Jahrom, Iran; Department of Medical Laboratory Sciences, School of Allied Medical Sciences, Iran University of Medical Sciences, Tehran, Iran

Dear Editor-in-Chief

COVID-19 is a newly emerged virus that has caused serious concerns for people around the world. The disease is the first infectious disease pandemic in the 21st century. To fight it, lockdown, social distancing, and staying at home strategies were implemented [1].

The COVID-19 pandemic has also affected educational systems around the world so that many students had to attend online classes. Online classes were one of the solutions to decrease the risk of infection [2]. Through this, learners and instructors used online platforms and screens (laptop, tablet, mobile phone, etc.) for long hours to attend their classes. There were also changes in lifestyle. To prevent COVID-19 infection, people had to stay at home and limit their social interactions to online platforms and social media (WhatsApp, Telegram, Instagram, and so on). Long hours of using displays increases the risk of sight disorders and refractive errors.

Myopia is a common refractive error in many countries. Refractive errors represent about one half of visual impairments and the second cause of functional blindness. They can degrade quality of life through affecting function, mental state, beauty, and financial pressure. In addition, refractive errors are more prevalent than other visual impairments during years lived with disability (YLDs). The errors can lead to loss of performance, function, job, and educational achievement if remain untreated [3]. Watching TV, computer games, using laptops, tablets, mobile phones and the like increase the risk of Myopia. There is a negative relationship between Myopia and the time spent outside home [4,5].

The risk of developing Myopia is higher during COVID-19 pandemic as many like students, university students, teachers, some professions, and many others must use online platforms to work. In addition, lockdown and spending long hours at home increase the time spent with mobile phones, laptops, TV, video games, and so on. Therefore, Myopia is one of the challenges that might appear in human societies following the pandemic. Given the importance of the disorder and its effect on the quality of life, there is a need for timely intervention and educational plans to inform the public. Along with public vaccination and booster injections, the ground for the presence of people in social and educational fields should be provided to promote social interactions in society. Parents are also recommended to spend a few hours everyday outside with their children while observing health codes such as using personal protection equipment including facial masks.

## Ethical Approval

This is a Commentary study and does not require Ethical Approval.

## Funding

This research did not receive any specific grant from funding agencies in the public, commercial, or not-for-profit sectors.

## Author contribution

RK: Idea design, writing—original draft preparation; FR: writing—review and editing; FD: writing—review.

## Registration of research studies

This is a commentary study and does not need registration of research studies.

## Guarantor

I accept the liability of the scientific integrity of the manuscript contents.

## Consent

This is a commentary study and does not require Consent.

## Declaration of competing interest

The authors declare that there is no conflict of interest.
